# Readability of Online Information on Core Decompression of the Hip for Avascular Necrosis

**DOI:** 10.7759/cureus.50298

**Published:** 2023-12-10

**Authors:** Aathir Ahmed, John Mahon, Ahmed Karkuri

**Affiliations:** 1 Orthopaedics, Royal College of Surgeons in Ireland, Dublin, IRL; 2 Orthopaedic Surgery, Sligo University Hospital, Sligo, IRL

**Keywords:** orthopaedics, readability, health literacy, avascular necrosis (avn), core decompression

## Abstract

Introduction

Avascular necrosis (AVN) of the femoral head is a type of osteonecrosis, which is caused by the disruption of blood flow to the proximal femur, resulting in osteocyte death. Regression of the disease is rare, and most patients will ultimately progress to having a total hip arthroplasty performed. Early diagnosis of AVN allows treatment options beyond total hip arthroplasty. One such procedure described is core decompression of the femoral head.

Health literacy is defined as the ability to make health decisions in the context of everyday life. It has been shown that lower levels of health literacy are associated with higher complication rates. It has been recommended that patient information documents are written at a reading grade level (RGL) no higher that the sixth grade to help with health literacy.

Methods

Twenty-nine websites containing information on core decompression were identified, and the online readability software WebFX (Pennsylvania, USA) was used to carry out analysis on readability. This software was able to generate a Flesch reading ease score (FRES) and an RGL for each website. The search was carried out in the Republic of Ireland.

Results

The mean FRES score was 48.8 (standard deviation (SD) +/-15.3), which categorizes the data as “difficult to read.” The mean RGL was 8.46 (SD +/-2.34), which is higher than the recommended target.

Conclusion

This study has shown that the material on the Internet regarding core decompression is above the recommended readability levels for the majority of patients. This aligns with results from similar studies that have assessed the readability of online patient information. Given these outcomes, it is imperative for physicians to take an active role in curating and delivering information to their patients, ensuring that it is comprehensible. This approach aims to empower patients with a clearer understanding of core decompression, enabling them to make more informed decisions about their health.

## Introduction

Avascular necrosis (AVN) of the femoral head is a type of osteonecrosis, which is caused by the disruption of blood flow to the proximal femur, resulting in osteocyte death. AVN may occur in the setting of either traumatic or non-traumatic ischaemia to the femoral head. The most common aetiologies of AVN of the femoral head are corticosteroids, fractures that disrupt the femoral blood supply, dislocations of the hip joint, and alcohol abuse [1].

AVN typically affects relatively young and active individuals between the ages of 20 and 40 years. Regression of the disease is rare, and most patients will ultimately progress to having a total hip arthroplasty performed [2]. In the United Kingdom, AVN is the third most common indication for total hip arthroplasty in patients under 50 years of age [3].

Early diagnosis of AVN allows treatment options beyond total hip arthroplasty. Core decompression (CD) of the femoral head has been described as one such treatment; this is intended to reduce intraosseous pressure and promote vascular inflow by drilling holes into the femoral head.

A study carried out by Ficat et al. followed 133 hips with AVN that underwent CD; it was found that 90% of patients with grade 1 and 2 AVN had good results, with minimal disease progression after a mean follow-up of 9.5 years [4].

CD is recommended as the first-line treatment for patients with early AVN. Multiple augmentation techniques with CD have been described to try and improve outcomes [5]. This is a complex procedure with ongoing surgical development and is not without surgical complications [6].

During the initial consultation, it can be difficult to explain the procedure to patients in a manner that is easy to understand, which may lead to confusion or being overwhelmed with information. Patients will often research their surgical procedure on the Internet to get a better understanding of the procedure itself, along with the expected postoperative recovery [7].

Hence, it is important that the information available online regarding surgical procedures is easily accessible and written at a level that facilitates adequate health literacy.

Health literacy is defined as the ability to make health decisions in the context of everyday life. Due to the unfamiliar medical vocabulary, patients may run into issues with health literacy [8]. It has been shown that lower levels of health literacy are associated with higher complication rates and re-hospitalization [9,10].

Using simple vocabulary, the inclusion of graphs and pictures instead of long lines of text and providing information at an appropriate grade level can improve health literacy, which is an independent and modifiable risk factor for postoperative complications [11].

The American population has an average reading grade level between the seventh and eight grade [12]. The US Department of Health and Human Services has recommended that patient information documents are written at a reading grade level (RGL) no higher than the sixth grade to help with health literacy [13]. However, the literature has proven that most online patient information is written well above recommended levels [13-15].

An extensive literature review of the MEDLINE database was carried out and found no previously published papers describing the accessibility of information on CD of the femoral head for AVN. The aim of this study is to analyze the readability of the online information regarding CD for AVN.

## Materials and methods

The search was carried out in the Republic of Ireland. Two search engines, Google and Bing, were accessed on a single day in August 2023, searching the term “hip core decompression for avascular necrosis.” For both search engines, the first three pages were evaluated, and a total of 73 websites were identified (n=73). The limit of three pages was set as previous studies demonstrated that most people do not look past the second page, with the majority only assessing the first page of a search engine [16]. The figures of results for each search engine are included in Table 1.

**Table 1:** Results of search terms

Search engine	Search term	Number of results
Google	Hip core decompression for avascular necrosis	92,000
Bing	Hip core decompression for avascular necrosis	163,000

**Figure 1:**
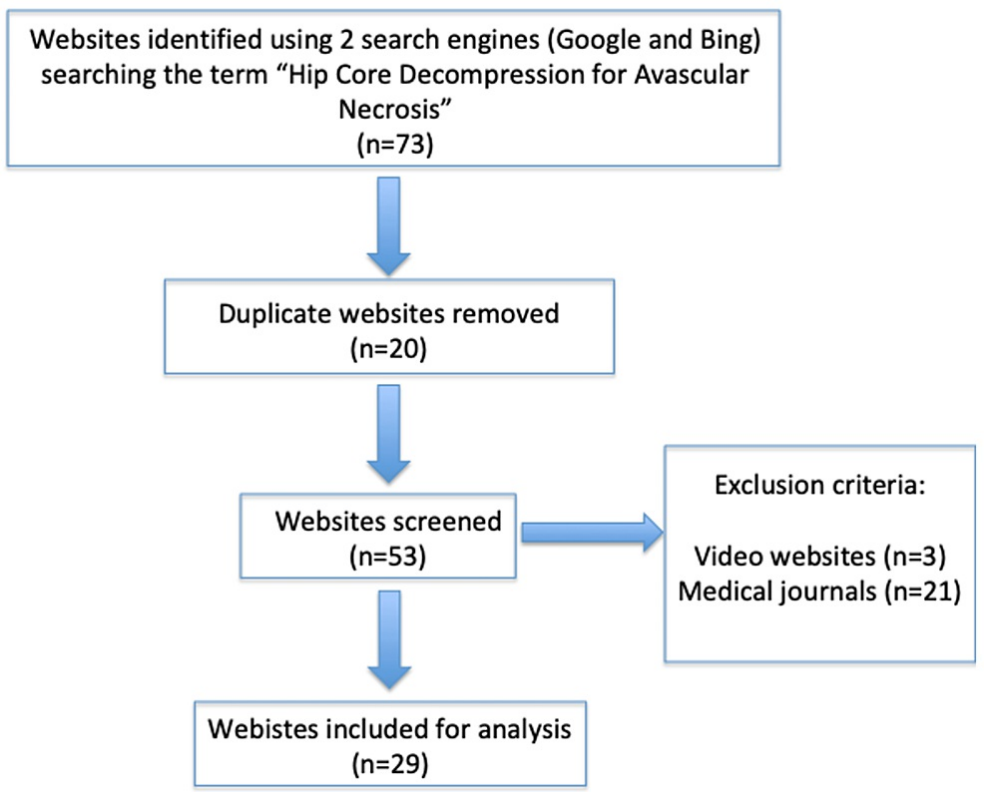
Flowchart of the inclusion methodology

We first removed duplicate websites (n=20), and the remaining (n=53) were accessed and assessed based on the exclusion criteria. Websites requiring a log-in, medical journals, and websites composed mostly of videos were excluded. This left us with a total of 29 websites, which met the inclusion criteria to undergo further in-depth analysis. A flow chart of the methodology is shown in Figure 1.

The following categories were used to classify the 29 websites: physician, non-physician, academic, commercial, media and news, social media, and non-specified. Websites affiliated with a university or teaching hospital were classified as “academic.” Physician websites referred to private websites run by doctors in private practice, while non-physician websites were linked with members of the multidisciplinary team, such as physiotherapists. Commercial websites were associated with the marketing of a product. A list of all included websites is referenced for review in Table 2.

**Table 2:** List of websites included for analysis

List of websites
https://manhattansportsdoc.com/hip-core-decompression-surgery-manhattan-new-york-city-ny/
https://www.leicesterhipandknee.co.uk/core-decompression-for-avascular-necrosis-of-the-hip.html
https://www.fischerjoints.com/core-decompression-for-avascular-necrosis-hip.html
https://www.orthopaedicsurgery.uci.edu/core-decompression-for-avascular-necrosis-of-the-hip-orthopaedic-irvine-newportbeach-california.html
https://orthoinfo.aaos.org/en/diseases--conditions/osteonecrosis-of-the-hip
https://www.drandrewdutton.com/blog/core-hip-decompression-avascular-necrosis/
https://www.uhcprovider.com/content/dam/provider/docs/public/policies/oxford/core-decompression-avascular-necrosis-ohp.pdf
https://www.cortho.org/hip/avascular-necrosis/core-decompression-avn-hip/
https://www.aetna.com/cpb/medical/data/700_799/0753.html
https://www.caringmedical.com/prolotherapy-news/avascular-necrosis-2/
https://houstonsportsortho.com/core-decompression-hip-doctor-sugar-land-pearland-houston-tx/
https://www.yoummd.com/arthroscopic-assisted-core-decompression-of-the-hip-hip-arthroscopy-surgeon-new-york.html
https://www.arthrex.com/hip/core-decompression
https://www.stlosm.com/core-decompression-avascular-necrosis-of-hip-orthopedics-sports-medicine-specialists-creve-coeur-missouri.html
https://davidslattery.com/hip-conditions/hav-osteonecrosis/treatment/
https://www.mayoclinic.org/diseases-conditions/avascular-necrosis/diagnosis-treatment/drc-20369863
https://www.vanthielmd.com/conditions-treatments/hip-arthroscopy-center/avascular-necrosis-of-the-hip/
https://www.corralesadvancedjoints.com/core-decompression-avascular-necrosis-hip.html
https://henrybackemd.com/avascular-necrosis/
https://stanfordhealthcare.org/medical-conditions/bones-joints-and-muscles/avascular-necrosis/treatments/core-decompression.html
https://en.wikipedia.org/wiki/Avascular_necrosis
https://www.aapc.com/codes/webroot/upload/general_pages_docs/document/Core_Decompression_For_Avascular_Necrosis.pdf
https://www.yalemedicine.org/conditions/avascular-necrosis-of-the-hip
https://www.dukehealth.org/treatments/orthopaedics/avascular-necrosis
https://www.physio-pedia.com/Avascular_Necrosis
https://patientslounge.com/procedures/Experiencing-Avascular-Necrosis-and-Total-Hip-Replacement
https://my.clevelandclinic.org/health/diseases/14205-avascular-necrosis-osteonecrosis
https://www.newyorkhipknee.com/faqs/avascular-necrosis-faqs/
https://www.orthospecialist.co.uk/core-decompression-for-avascular-necrosis-of-the-hip-mr-yegappan-kalairajah.html

The online readability software, WebFX (Pennsylvania, USA), was used to carry out the analysis of the included websites. This software was able to generate a Flesch reading ease score (FRES) and an RGL for each website [17].

The FRES is based on a 0-100 scale. It is generated by an algorithm that considers factors, such as syllable count, sentence length, and the use of complex words, to gauge the overall readability and complexity of a piece of text. A high score means that the text is easier to read. Low scores suggest that the text is complicated and difficult to understand. A value between 60 and 80 should be easy for a 12- to 15-year-old to understand. [17] A breakdown of the FRES scoring system is shown in Table 3 [18].

**Table 3:** Flesch reading ease score (FRES) Reference: [18]

Score	School level (US)	Notes
100-90	5th grade	Very easy to read; easily understood by an average 11-year-old student
90-80	6th grade	Easy to read; conversational English for consumers
80-70	7th grade	Fairly easy to read
70-60	8th and 9th grades	Plain English; easily understood by 13- to 15-year-old students
60-50	10th to 12th grades	Fairly difficult to read
50-30	College	Difficult to read
30-10	College graduate	Very difficult to read; best understood by university graduates
10-0	Professional	Extremely difficult to read; best understood by university graduates

The RGL is a measure commonly used to assess the readability of text in the context of the American education system. The idea is to represent the difficulty of the text in terms of the number of years of formal education a person would typically need to easily understand it on the first pass. A good target for readability to maximize comprehension is the 7th grade [17].

## Results

From the initial 73 websites identified during the search, 29 met the inclusion criteria to undergo readability analysis using the software WebFX. This included 15 physician websites, seven academic websites, four non-specified websites, one commercial website, one non-physician website, and one social media website.

The mean FRES score was 48.8 (standard deviation (SD) +/-15.3), which categorizes the data as “difficult to read” (Table 3). Thirteen (45%) of the websites had a FRES score less than 50, indicating that they would require the reader to be at college level or higher to read and understand the material. In this study, the non-physician website had the lowest mean FRES scores, indicating that it was the hardest category for readers online, while the social media website had the highest mean FRES score, giving it the easiest to read ranking among the others. This is outlined in Table 4.

**Table 4:** Breakdown of FRES and RGL scores based on website category FRES: Flesch reading ease score, RGL: reading grade level

	Type of website	N	Mean	Median	SD	Minimum	Maximum
FRES	Physician	15	47.49	49.9	12.33	15.8	68
	Academic	7	49.9	51.6	5.74	40.6	57
	Non-specified	4	50.13	50.45	36.95	9.9	89.7
	Commercial	1	51	51	NaN	51	51
	Non-physician	1	46.4	46.4	NaN	46.4	46.4
	Social media	1	56.4	56.4	NaN	56.4	56.4
RGL	Physician	15	8.8	8.3	2	6	13.2
	Academic	7	8.26	8.2	1.55	6.2	11
	Non-specified	4	7.42	7.65	4.92	1.7	12.7
	Commercial	1	7.2	7.2	NaN	7.2	7.2
	Non-physician	1	8.8	8.8	NaN	8.8	8.8
	Social media	1	9.9	9.9	NaN	9.9	9.9

The mean RGL was 8.46 (SD +/-2.34), which is higher than the recommended target. Seventeen (59%) of the websites had an RGL of 8 and higher, which is above the recommended level for readability. As outlined in Table 4, the social media category had the highest RGL, while the commercial website category had the lowest RGL.

Both the FRES and RGL scores indicate that the majority of websites are written at a level that make them difficult to read and hence reduce the readers ability to easily comprehend the information provided. A breakdown of the mean FRES and RGL scores for each category can be found in Table 4. 

**Figure 2:**
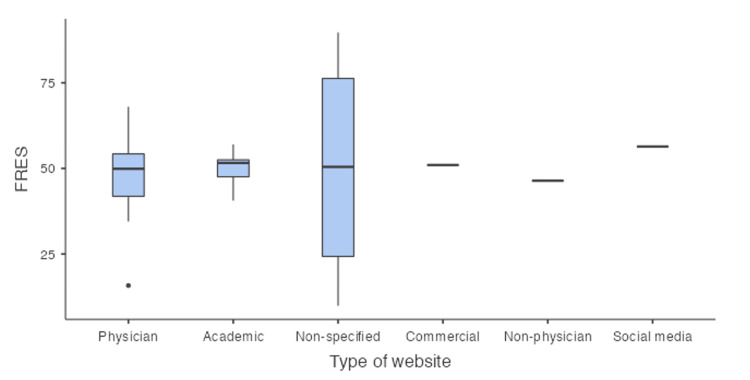
Box-plot representation of FRES FRES: Flesch reading ease score

**Figure 3:**
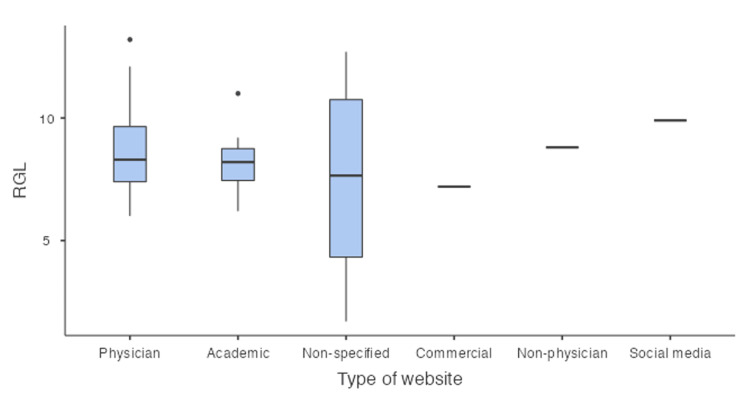
: Box-plot representation of RGL RGL: reading grade level

The box-plot representations of the FRES and RGL scores are illustrated in Figure 2 and Figure 3, respectively

## Discussion

AVN of the femoral head is a progressive disease that normally leads to collapse of the femoral head [1]. It is a disabling condition that affects adults aged 30 to 50. The goals of treatment for early disease are pain relief, preservation of the femoral head function, and delay in time to total hip arthroplasty.

CD is a minimally invasive procedure that alleviates pressure and ischemia in the femoral head and removes necrotic bone impeding revascularization of the femoral head. There are many novel variations to CD surgery combining it with bone grafting, stem cell transplantation, and bone marrow aspiration, among others [19].

Given the variety of treatment options available to patients, it can be challenging for physicians to explain this information in a concise manner during a consultation. As a result, there has been an increasing trend towards patients accessing the internet to carry out their own research for a “second opinion.”

However, much of the information available online is unregulated, which may result in misinformation being distributed. Therefore, it is important that the patient information available on the Internet is easily accessible, accurate, and written at a level that is easily understood by the general population.

The FRES scoring system was the validated readability tool that was used in our study. This incorporates the average sentence length and average syllables per word into a formula, which calculates a score from 0 to 100. The higher the score, the easier the text is to read. Authors can optimize the readability of text, guided by this formula, using shorter sentences and shorter words [20]. In our study, the mean FRES score was 48.8, indicating that the patient information available online is difficult to read overall.

The second assessment of readability was carried out using the RGL. Although different sources have recommended different reading grade levels for online medical information, the general consensus is that an RGL between the sixth and eight grade is an acceptable level [21,22]. In our study, the mean RGL was 8.46, with 59% being higher that the eight grade and only 10% being at sixth grade or lower.

The main finding in our study is that the information available online regarding CD is written at a level that is too difficult to read for the general population. Our results are consistent with similar studies performed to assess the readability of online information for patients [13,15,16,23].

We acknowledge that there are several limitations to this study. First, the search of terms was carried out at a single time point, which provides a cross-sectional snapshot of information available online. Second, the search was carried out in a single country (Republic of Ireland), which may skew representation of national websites. Lastly, although the two most common search engines were used, this does not account for other sources patients may use to access information.

## Conclusions

The findings of this study highlight that the material related to CD available on the Internet surpasses the recommended readability levels for the majority of the population. This aligns with results from similar studies that have assessed the readability of online patient information. Given these outcomes, it is imperative for physicians to take an active role in curating and delivering information to their patients, ensuring that it is comprehensible. This approach aims to empower patients with a clearer understanding of CD, enabling them to make more informed decisions about their health. By offering vetted information, healthcare providers contribute to fostering a patient-centered approach and facilitating improved communication between medical professionals and patients.
